# High‐Throughput Geocoding to Assess Short‐Term Air Pollution in Correlation With Mortality in a French Cancer Patient Cohort

**DOI:** 10.1002/ijc.70589

**Published:** 2026-06-07

**Authors:** Loice Pokam, Joséphine Bocquet, Nadia Hégarat, Julie Perlbarg‐Samson, Thomas Balezeau, Maud Milder, Bastien Rance, Hector Countouris, Julien Guerin, Flavien Gilles, Jean Gaudart, Nicolas Girard, Clémence Basse

**Affiliations:** ^1^ Thorax Institute Curie‐Montsouris Hôpital Institut Curie Paris France; ^2^ Department of Medical Informatics Hôpital Européen Georges Pompidou, Assistance publique–Hôpitaux de Paris Paris France; ^3^ Medical Informatics Department Institut Curie Paris France; ^4^ Data Department Institut Curie Paris France; ^5^ INSERM, Centre de Recherche des Cordeliers, UMRS 1138 Université de Paris, Université Sorbonne Paris Cité Paris France; ^6^ IRD, INSERM, SESSTIM, UMR1252, ISSPM, AP‐HM, BioSTIC, Biostatistics & ICT Unit Aix Marseille University Marseille France; ^7^ Santé Publique France, Department of Environmental Health French National Public Health Agency Saint Maurice France; ^8^ Paris Saclay Campus University Versailles Saint Quentin Versailles France

**Keywords:** air pollution, cancer, exposome, geomatic, mortality

## Abstract

Air pollution significantly affects human health and mortality, including cancer‐related deaths. However, methodological approaches and spatial precision often vary across studies. Implementing geomatic tools to investigate mortality in vulnerable populations such as cancer patients may enhance understanding of environmental impacts. This study aimed to assess the relationship between short‐term air pollution exposure and mortality among cancer patients in France. A retrospective cohort of 44,268 consecutive cancer patients (2017–2020) was analyzed using a geomatic framework at fine spatial granularity. Air quality data (PM_2.5_, PM_10_, NO_2_, O_3_, temperature) were linked to each patient. Principal component analysis (PCA) identified exposure groups, and a random forest algorithm was applied to predict mortality. Overall, 9% of patients died during follow‐up. Four clusters of patients with distinct air quality profiles were identified. Clusters with the highest particulate matter levels (PM_2.5_, PM_10_, NO_2_) showed increased mortality (12%–13%), whereas the cluster with the lowest pollution showed reduced mortality (8%) (chi‐square test, *p* < 0.001). The prediction algorithm achieved a recall of 80%. The main predictors of death included higher temperature and elevated PM_2.5_ within the previous 31 days, as well as older age, male sex, and thoracic cancer. Short‐term exposure to degraded air quality, captured at an infra‐communal scale, is associated with excess mortality among cancer patients. The identification of a 31‐day lag window for predictive algorithm highlights opportunities for targeted prevention and timely public health interventions.

## What's New?

Although chronic air pollution exposure is linked to lung cancer mortality, the short‐term effects of fluctuating air quality on overall cancer mortality remain uncertain. Here, the authors applied geomatics and machine‐learning models to retrospectively examine possible associations between 31‐day pollution exposure and mortality in a French cancer cohort. High‐resolution data reveal significantly elevated death rates among patients exposed to higher levels of particulate matter and nitrogen dioxide. Elevated PM_2.5_ and temperature were among the strongest mortality predictors. The findings identify a critical short‐term risk window and highlight the importance of fine‐scale environmental monitoring for vulnerable cancer populations.

## Graphical abstract

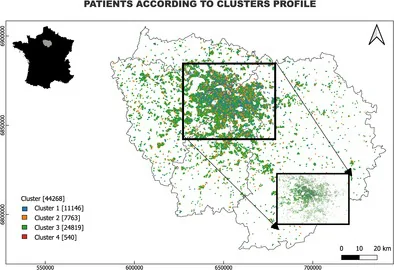

**Abbreviations**
API: application programming interface
AUC: area under the curve
BC: Before Christ
COPD: chronic obstructive pulmonary disease
DNA: deoxyribonucleic acid
EEA: European Environment Agency
EORTC: European Organisation for Research and Treatment of Cancer
EU: European Union
IARC: International Agency for Research on Cancer
ICD‐10: International Classification of Diseases, 10th revision
IRIS: Ilots Regroupés pour l'Information Statistique = “Aggregated Units for Statistical Information”
NIH: National Institutes of Health
NO_2_
: nitrogen dioxide
O_3_
: ozone
PCA: principal component analysis (a statistical method to reduce data dimensions)
PCs: principal components (PC1 and PC2)
PM_10_
: atmospheric particulate matter with a diameter of less than 10 μm
PM_2.5_
: atmospheric particulate matter with a diameter of less than 2.5 μm
ROC: receiver operating characteristic curve
SPECTA: clinical trial screening cancer patients for efficient clinical trial access
WGS84: World Geodetic System 1984
WHO: World Health Organization
ZIP: zone improvement plan

## 1 Introduction

Air pollution has been recognized as a potential threat to human health since the time of Hippocrates (circa 400 bc) [1]. Following the industrial revolution, the documentation of critical levels of air pollution occurred, although their health implications were not fully understood at the time [1]. Throughout the 20th century and beyond, numerous studies have explored the relationship between air pollution and human health. Chronic exposure to nitrogen dioxide (NO_2_)—a gas primarily generated by the combustion of fossil fuels—and fine particulate matter (PM_2.5_, particles with an aerodynamic diameter smaller than 2.5 μm, largely produced by the burning of gasoline, oil, diesel, and wood) has been linked to respiratory symptoms such as cough and sputum production [2]. Elevated NO_2_ concentrations are also associated with chronic bronchitis [2]. Additionally, several studies have reported that long‐term exposure to PM_2.5_ significantly increases the risk of asthma in both children and adults [3]. Higher PM_2.5_ concentrations have been shown to correlate with increased hospital admissions and emergency department visits for asthma and chronic obstructive pulmonary disease (COPD) [3]. Chronic exposure to ambient PM_2.5_ and ozone (O_3_, a gas formed under ultraviolet radiation) has also been linked to a range of diseases, including cardiovascular events, dementia [4], and certain cancers [5]. In line with these results, a recent study conducted in mainland China demonstrated a correlation between bronchiectasis mortality and short‐term (3‐day) exposure to PM_2.5_, PM_10_, and O_3_ [6]. The International Agency for Research on Cancer (IARC) has classified particulate matter, diesel exhaust, and overall outdoor air pollution as Group 1 carcinogens [7], with the strongest evidence supporting a positive association between air pollution—particularly PM_2.5_—and lung cancer [8].

Exposure to fine particulate matter (PM_2.5_) and ozone (O_3_) has also been linked with reduced life expectancy [9]. The World Health Organization (WHO) has established global air quality guidelines and continues to highlight the severe health risks associated with air pollution, which is estimated to cause millions of deaths annually [10, 11]. Evidence suggests that the long‐term effects of nitrogen dioxide (NO_2_) on mortality may be comparable to those of PM_2.5_ [12]. While numerous studies have demonstrated associations between air pollution exposure and cancer incidence, chronic exposure to PM_2.5_ has also been significantly associated with increased lung cancer mortality [8, 13].

Most existing research focuses on the long‐term effects of air pollution, whereas short‐term exposures remain less frequently studied [14]. A recent Brazilian study, conducted at the municipal level, examined acute PM_2.5_ exposure and its association with overall cancer mortality [15]. However, the short‐term impact of air quality fluctuations on cancer‐related deaths appears to be underreported and likely underestimated [14]. The optimal lag period preceding death for assessing short‐term air pollution effects remains uncertain. Reported lag windows in the literature vary widely, ranging from one to 5 days [6, 15, 16], up to 7 days [17], and in some cases covering a full month prior to death [18, 19]. Overall, most studies suggest a delayed response period during which short‐term exposure to air pollutants shows measurable effects on mortality [4, 20, 21, 22].

Cancer patients represent a uniquely vulnerable population that warrants particular attention due to their compromised health status. Institut Curie, a leading cancer center in France, aims to anticipate and address the emerging challenges faced by cancer patients through the use of innovative technological tools. Despite significant advances in understanding the health effects of air pollution, there remains a pressing need to refine methods for quantifying and characterizing air pollution–health relationships. This includes improving spatial resolution, expanding geographic coverage, and developing standardized analytical frameworks to ensure comparability across studies [23].

The objective of the present study is to examine the association between short‐term air pollution exposure (within a 31‐day lag period) and mortality in a French cohort of cancer patients. Using a geomatic framework and predictive algorithm tools, we analyzed high‐resolution environmental data, including PM_2.5_, PM_10_, NO_2_, and O_3_ concentrations at a spatial resolution of 50 × 50 m, and temperature data at a spatial resolution of 9 × 9 km.

## 2 Materiel and Methods

### 2.1 Geocoding Procedure

Geocoding was performed using the Addok application programming interface (API), developed by Etalab, the French interministerial task force responsible for open data management. Addok is an extensible, plugin‐based system designed specifically for metropolitan French postal addresses. It indexes postal address data and provides spatial coordinates (*x*, *y*) based on the World Geodetic System 1984 (WGS84). Once geocoding was completed, the resulting coordinates were stored in a dedicated file or database. A spatial join was then applied between the geocoded patient addresses and the IRIS (Ilots Regroupés pour l'Information Statistique) shapefile, allowing the integration of accurate geographic information. These spatial coordinates enabled the linkage between patients' postal addresses and environmental data, including air quality (PM_2.5_, PM_10_, NO_2_, O_3_) and temperature variables.

### 2.2 Patients and Clinical Data

This study was based on a retrospective cohort of cancer patients managed at Institut Curie, a leading French comprehensive cancer center. The study period spanned from January 1, 2017, to December 31, 2019. Patients were identified using Consore, a search engine developed by Unicancer for clinical data retrieval. Inclusion criteria were as follows: patients with at least 15 clinical documents in their medical records, to ensure continuous follow‐up at Institut Curie during the study period; patients diagnosed with a single cancer type, as defined by the International Classification of Diseases, 10th revision (ICD‐10) [24]; and patients with a documented vital status from the most recent available update within the study period.

The analysis was restricted to patients residing in the Paris metropolitan area, where high‐resolution air quality data were available, and to individuals who had not objected to the reuse of their medical data for research purposes. Collected clinical variables included the postal address, sex, age, tumor type, most recent clinical update during the study period, and vital status (alive or deceased).

### 2.3 Confounding Factors, Pairing and Reference Date

Confounding factors associated with death were identified prior to matching. Subsequently, patients who died were matched with patients who survived during the study period. The matching procedure used was the same as that typically used in case–control studies, although the present study was not a case–control analysis.

Each “control” patient (i.e., one who did not die during the study period) was assigned a reference date for air quality assessment corresponding to the death date of the nearest “case” patient (i.e., one who died during the period of interest). A single case could be matched to multiple control patients (1:N matching). This approach identifies the nearest neighbor based on the dimensions of the previously defined confounding variables. Reference dates for control patients were therefore determined based on the death dates of their matched cases.

### 2.4 Air Quality and Meteorological Data

Air quality data were provided by AIRPARIF, an official organization responsible for atmospheric monitoring in the Paris region [25]. The dataset included daily concentrations of major pollutants—PM_10_, PM_2.5_, NO_2_, and O_3_—delivered in raster format with a spatial resolution of 50 × 50 m. In addition, meteorological data were used to account for environmental variability. Hourly air temperature at 2 m above ground level was obtained, calculated by interpolation between the lowest model level and the Earth's surface while considering prevailing atmospheric conditions. These temperature data were retrieved through a client API communicating with the Copernicus ERA5‐Land dataset and subsequently aggregated to daily means. The resulting temperature rasters had a spatial resolution of 0.1 × 0.1° (approximately 9 × 9 km) [26].

### 2.5 Short‐Term Air Quality Data: Timepoints and Pollution Thresholds

A 31‐day lag period preceding each reference date was defined to assess short‐term exposure to air pollutants. Within this lag window, six specific timepoints were established: D‐1, D‐2, D‐3, D‐7, D‐15, and D‐31. For each patient and reference date, air quality measurements corresponding to these six timepoints were extracted for each of the environmental variables, including PM_10_, PM_2.5_, NO_2_, O_3_, and temperature.

At each timepoint, the recorded value represented either the maximum concentration of the pollutant observed within the defined period or the number of exceedance peaks occurring between the reference date and the given timepoint. For instance, NO_2_ D31 max corresponds to the highest NO_2_ concentration recorded during the 31 days preceding the reference date for a given patient.

### 2.6 Air Quality Thresholds

According to the WHO, the recommended daily average limits—which should not be exceeded for more than 3 days per year—are as follows: 15 μg/m^3^ for PM_2.5_, 45 μg/m^3^ for PM_10_, 25 μg/m^3^ for NO_2_, and 100 μg/m^3^ for O_3_ (measured as the 8‐h daytime maximum) [27].

The European Environment Agency (EEA) also defines air quality limit values. For chronic exposure, daily PM_10_ concentrations above 50 μg/m^3^ should not be exceeded for more than 35 days per year, and hourly NO_2_ concentrations above 200 μg/m^3^ should not be exceeded more than 18 times annually. Acute O_3_ pollution is defined as a daily 8‐h daytime average exceeding 180 μg/m^3^. The EEA does not provide a short‐term exposure threshold for PM_2.5_ [28]. French regulations follow EU (European Union) average values to establish information thresholds for pollution episodes. In this study, these thresholds were used to define acute pollution events, according to either WHO or EEA guidelines [29].

### 2.7 Statistics, Principal Components Analysis, and Clustering

A principal component analysis (PCA) was conducted. The objective was to reduce complexity of the dataset composed of air quality characteristics and temperature characteristics across different timepoints. We then performed a non‐supervised clustering analysis of the dataset to identify distinct exposure profiles among the patient cohort. The algorithm used was *K*‐means clustering, applied to the necessary principal components (PCs) that explained 99% of the total variance. The optimal number of clusters (*k*) was determined using the Elbow method. The analysis of inertia curves indicated that *k* = 7 showed lower inertia but with more fragmented groups. However, after iterative analysis, *k* = 4 was determined to provide a clearer distinction of four clusters of pollution exposure, which was more interpretable for the present project.

Based on the literature, potential confounding factors such as age, sex, and pathology were identified. Bivariate analyses were subsequently performed to assess their association with death: chi‐square tests for categorical variables, and Mann–Whitney *U* or Student's *t*‐tests for continuous variables. Factors showing significant associations (according to *p* values and effect size thresholds) were retained as confounders. These variables were then included in a logistic regression model to determine whether they represented independent risk factors for death. This multivariate approach allowed us to estimate adjusted odds ratios, account for the influence of demographic and clinical determinants, and thus better isolate the specific contribution of air quality variables.

Later on, the spatial distribution of patients according to their cluster profile was analyzed using Moran's *I* to assess global autocorrelation. To further explore local spatial patterns, Getis‐Ord Gi* statistics were applied, identifying specific hotspots and coldspots of pollution.

### 2.8 Random Forest Method and Predictive Algorithm for Death

To develop a predictive model for mortality, we employed the random forest algorithm. This method relies on an ensemble of multiple decision trees. Each tree, constructed from a random subset of features and samples, produces an individual classification, and the final prediction is typically determined by majority voting across all trees. Because our dataset was imbalanced—with substantially fewer death cases than control cases (surviving patients during the study period)—several resampling strategies were applied to address this issue and improve the model's ability to identify the minority class of death cases.

We used 80% of the dataset for training the predictive algorithm and the remaining 20% for testing and validation. This partitioning enabled the assessment of the algorithm's generalization performance on unseen data. To ensure robustness, cross‐validation was applied during the training phase, thereby reducing the risk of overfitting. Model performance was evaluated using the area under the receiver operating characteristic curve (ROC AUC) to verify that the algorithm performed better than random classification and the recall metric (the proportion of true positive predictions), as accurately identifying death cases was considered more critical than achieving higher overall accuracy.

### 2.9 Software

The software used was QGIS for mapping and cartographic visualization, particularly for handling raster datasets, spatial analysis, and representation of air quality and temperature data. Python was used for data processing, statistical analysis, prediction, with libraries such as pandas, NumPy, scikit‐learn, statsmodels, seaborn, and matplotlib for visualization of the results.

## 3 Results

### 3.1 Patients' Characteristics

In our final cohort 44,268 patients were included (flow chart—Figure S1). Males represented 20% of the cohort (*n* = 8854). The median age at the beginning of the study was 64 years old [16–100]. Forty‐seven percent of patients were aged > 65 years, and 80% were aged > 50 years. Primary cancer sites were as follows: breast (64%), urologic tract (9%), gynecologic (4%), hematologic (4%), digestive tract (4%), thoracic (3%), and head and neck cancers (3%) (Table 1). Regarding patient geographic origin, most patients came from the city of Paris itself (24%), and from the western Parisian departments Haut‐de‐Seine (23%) and Yvelines (19%), Figure S2.

**TABLE 1:** Patients' characteristics.

Characteristics	Number of patients (%)
Total population	44,268 (100%)
Age at the beginning of the period (January 1st 2017)	
16–50 years old	8809 (20%)
51–65 years old	14,862 (34%)
66–80 years old	15,964 (36%)
> 81 years old	4633 (10%)
Gender	
Female	35,414 (80%)
Male	8854 (20%)
Malignancy origin	
Breast cancer	28,433 (64%)
Urologic cancer	4022 (9%)
Gynecologic cancer	1921 (4%)
Hematologic cancer	1824 (4%)
Digestive cancer	1607 (4%)
Thoracic cancer	1516 (3%)
Head and neck cancer	1088 (3%)
Sarcoma	873 (2%)
Ophthalmologic cancer	847 (2%)
Dermatologic cancer	811 (2%)
Endocrinologic cancer	804 (2%)
Neurologic cancer	392 (1%)
Other cancer	130 (0%)

### 3.2 Determination of Clusters of Air Quality Exposures—PCA

**FIGURE 1:**
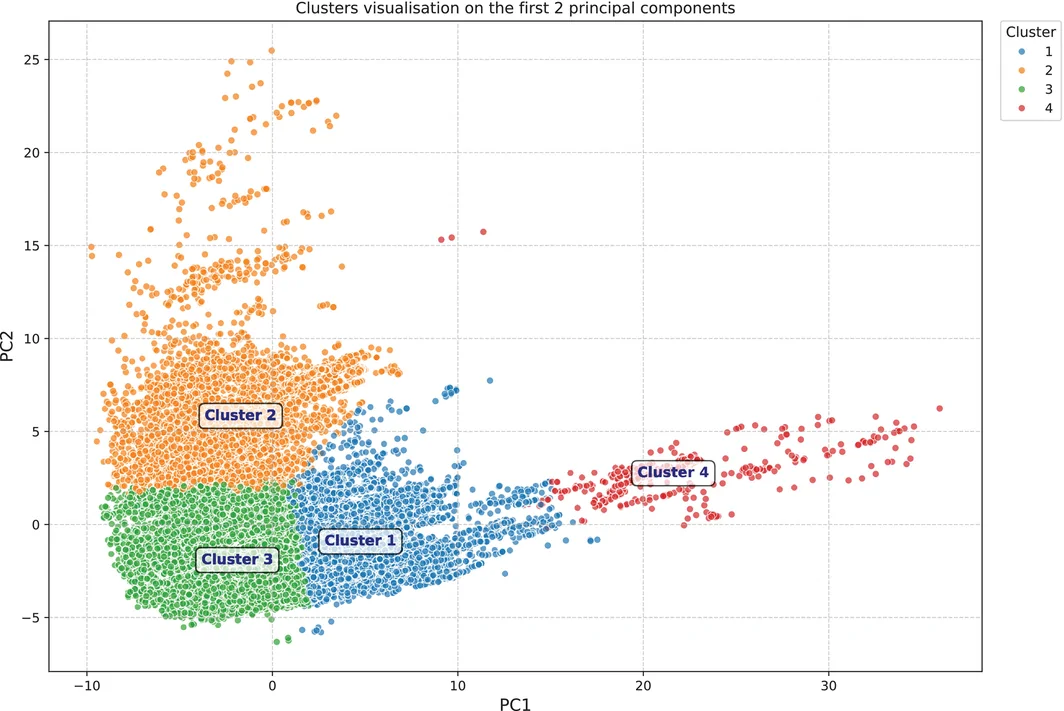
Clusters of patients by air quality exposure.

In the PCA that was implemented, to better visualize the data, we plotted the patients on a plane defined by the first two PCs (PC1 and PC2), which together explained 44% of the total variance of the dataset (Table S1). PC1 aggregated particulate matter data (PM_2.5_, PM_10_, NO_2_), whereas PC2 aggregated temperature and O_3_ data (Figure S3). The projection of the clusters in the PCA plane (PC1 vs. PC2) allowed a clear distinction of four patient groups with distinct air quality exposures, Figure 1. The proportion of patients in each air quality cluster was as follows: Cluster 1, 25% (*n* = 11,146); Cluster 2, 18% (*n* = 7763); Cluster 3, 56% (*n* = 24,819); and Cluster 4, 1% (*n* = 540). Cluster 1 included patients exposed to high particular matter levels (PM_2.5_, PM_10_, and NO_2_) but low temperatures and O_3_ exposure; Cluster 2 included patients exposed to high temperatures and O_3_ levels, but lower levels of particulate matters; Cluster 3 included patients exposed to lower temperatures and lower O_3_ levels, and lower particulate matters concentrations (the best air quality in the cohort); and finally, Cluster 4 included patients exposed higher levels of particulate matters, high O_3_, and high temperatures (the worst conditions compared to other clusters). The median level of PM_2.5_ was 35, 23, 22, and 47 μg/m^3^ in Cluster 1, Cluster 2, Cluster 3, and Cluster 4 respectively. The median level of PM_10_ was 42, 36, 30, and 63 μg/m^3^ in Cluster 1, Cluster 2, Cluster 3, and Cluster 4. The median level of NO_2_ was respectively 99, 84 and 79, and 111 μg/m^3^ in Cluster 1, Cluster 2, Cluster 3, and Cluster 4 respectively (Table 2). Air quality data were above WHO guidelines in all clusters for PM_2.5_, but only 50% of clusters regarding EEA guidelines. Cluster 4 exceeded PM_10_ threshold according to the WHO and EEA guidelines. NO_2_ levels were never above guidelines (neither WHO's nor EEA's). Cluster 2 was above WHO's threshold for O_3_ levels, but not for EEA's threshold, Table 2.

**TABLE 2:** Main air pollution characteristics in the four clusters.

Air pollution		Cluster 1	Cluster 2	Cluster 3	Cluster 4
PM_2.5_	PM_2.5_				
	Mean/median	37/35	25/23	24/22	51/47
	[Range] in μg/m^3^	[15–86]	[9–40]	[7–74]	[22–85]
	PM_2.5_ peak				
	Above threshold WHO/EEA	Yes/yes	Yes/no	Yes/no	Yes/yes
PM_10_	PM_10_				
	Mean/median	46/42	35–36	33–30	55–63
	[Range] in μg/m^3^	[20–98]	[15–55]	[13–78]	[47–96]
	PM_10_ peak				
	Above threshold WHO/EEA	No/no	No/no	No/no	Yes/yes
NO_2_	NO_2_				
	Mean/median	102/99	85/84	82/79	110/111
	[Range] in μg/m^3^	[37–174]	[12–180]	[14/181]	[57–138]
	NO_2_ peak				
	Above threshold WHO/EEA	No/no	No/no	No/no	No/no
O_3_	O_3_				
	Mean/median	92/86	156/152	108/91	81/82
	[Range] in μg/m^3^	[13–191]	[106–229]	[21–229]	[60–178]
	O_3_ peak				
	Above threshold WHO/EEA	No/no	Yes/no	No/no	No/no
T°	Temperature				
	Mean/median	14/12	24/25	19/19	10/9
	[Range] in °C	[0–29]	[2–33]	[0–33]	[5–33]

*Note:* Air quality data are the short‐term data in the 31‐day window before the reference date for each patient in the clusters.

Abbreviations: EEA, European Environment Agency; WHO, World Health organization.

**FIGURE 2:**
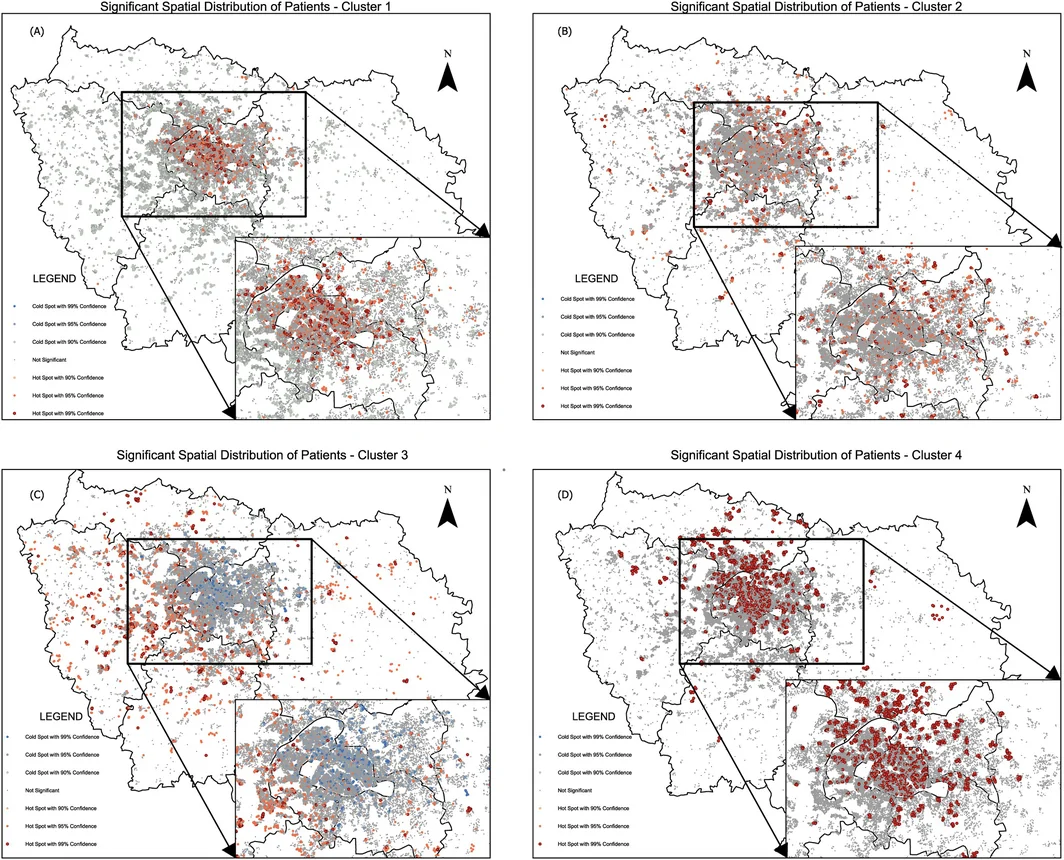
Significant spatial distribution of patients by cluster: (A) Cluster 1. (B) Cluster 2. (C) Cluster 3. (D) Cluster 4.

Patients in each cluster (exposed to similar pollution profiles) were nonrandomly grouped together, according to Moran score (Moran index was *I* = 0.046, and Moran score was *z* = 20, *p* < 0.001), graphical abstract. This suggests a strong link between geographic localization of patients' home addresses and air quality on these specific spots. We performed an additional Getis‐Ord Gi analysis to detect hot and cold spots within each cluster (with significant association with air quality data in the cluster). We found that patients in Cluster 1 were mostly living in Paris itself and in the close belt of the city of Paris; patients in Cluster 2 were mostly living at a mild distance from Paris (not in the center) and in the closest departments jouxting the city of Paris; patients in Cluster 3 were living the farthest from the center of Paris; and patients in Cluster 4 were grouped in islets in the close departments adjacent Paris in the north and north–west (Figure 2A–D).

### 3.3 Occurrence of Death

Death occurred in 3990 patients (9% of the cohort), and 2506 (63%) were older than 65 years. Among men, death occurred in 1405 out of 8854, or 16%. The mortality rates in tumor pathology subgroups were in decreasing order: 37% in thoracic (563/1516), 26% in digestive (411/1607), 18% in head and neck (199/1088), 15% in sarcoma (134/873), 14% in gynecologic (274/1921), 9% in urologic (373/4022), 10% in hematologic (188/1824), 8% in other (295/2984), and 5% in breast tumors (1553/28433).

Bivariate analysis indicated that significant confounding factors for death were age at the start of the study, tumor pathology, and sex. The odd ratios according to age groups were in decreasing order: 7.8 for > 81 years old [95% CI: 5.9–10.4], 2.3 for 66–80 years old [2.2–3.8], 1.9 for 51–65 years old [1.4–2.5], 1.7 for 36–50 years old [1.3–2.3], compared to the 16–36 years old group. The odd atios for death above one were among tumor pathologies: 2.9 for thoracic [95% CI: 1.8–4.9]; 1.7 for gastric [1.0–2.3]; 1.1 for neurologic [0.6–1.9]; 1.0 for gynecological [0.6–1.7]; and 1.0 for sarcoma [0.6–1.7] tumor types, compared to other pathologies. The odd ratio for death in men was 1.5 [95% CI: 1.3–1.6] compared to women. Normalized by patients per cluster, death occurred in Cluster 1 in 12% of patients, in Cluster 2 in 9%, in Cluster 3 in 8%, and in Cluster 4 in 13%. To assess whether clustering results and the distribution of deceased patients in each cluster were not due to random variation, we performed the chi‐square test of independence between cluster and death status, which reported a *p* value < 0.001, indicating a significant difference in death rates across clusters. Cluster 1 and Cluster 4, with the highest levels of particulate matters, showed excess mortality. Whereas the less polluted Cluster 3 presented a reduced mortality rate (Figure 1, Table 2).

### 3.4 Predictive Algorithm for Death Associated With Air Quality Data

**FIGURE 3:**
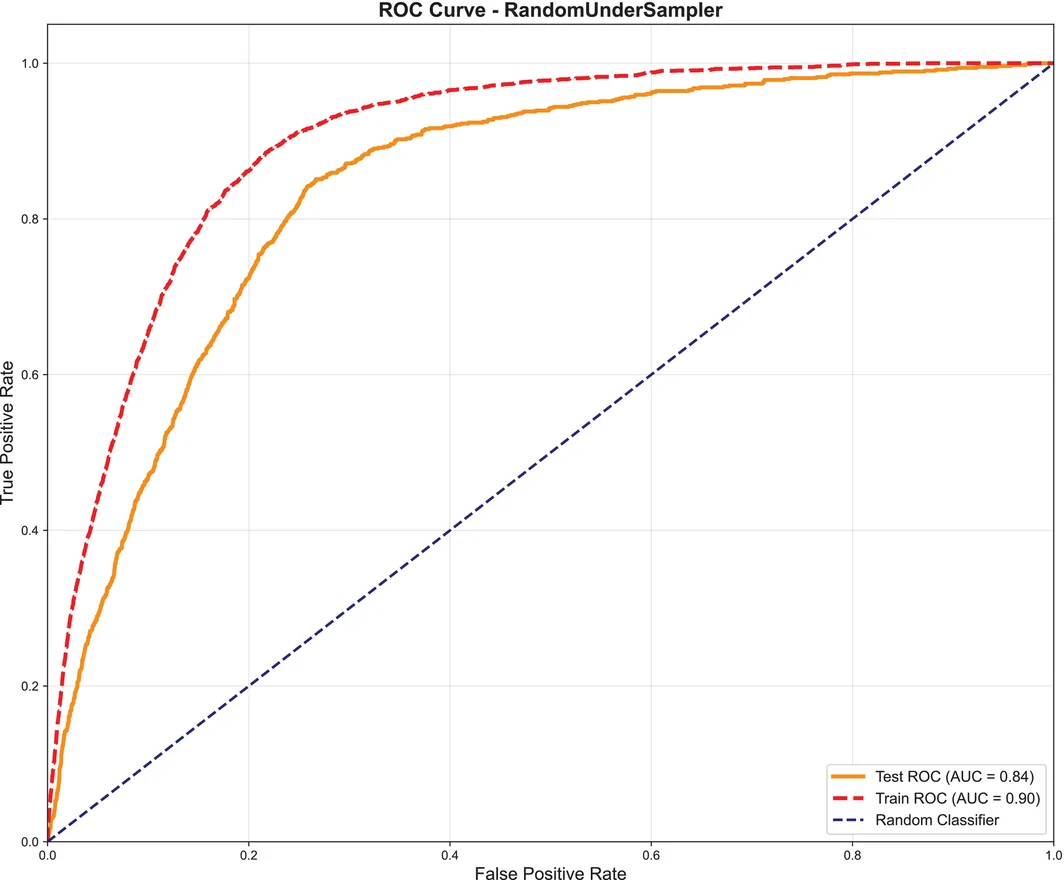
ROC curve.

After defining the confounding factors for death (sex, age, and tumor pathology), we developed a predictive algorithm to forecast the occurrence of death in our cohort based only on air quality variables (independent risk factors were excluded to reduce bias). The machine learning process used 80% of the dataset for training (*n* = 35,414 patients), and we tested the predictive algorithm on 20% of the dataset (*n* = 8854 patients). Among the different methods for data balancing, the best one in terms of recall turned out to be the “random under sampler”: the model predicted patient death with 78% accuracy, 80% recall, F1‐score of 0.41, and area under the curve (AUC) of 0.85 (Figure 3, and Figure S4).

**FIGURE 4:**
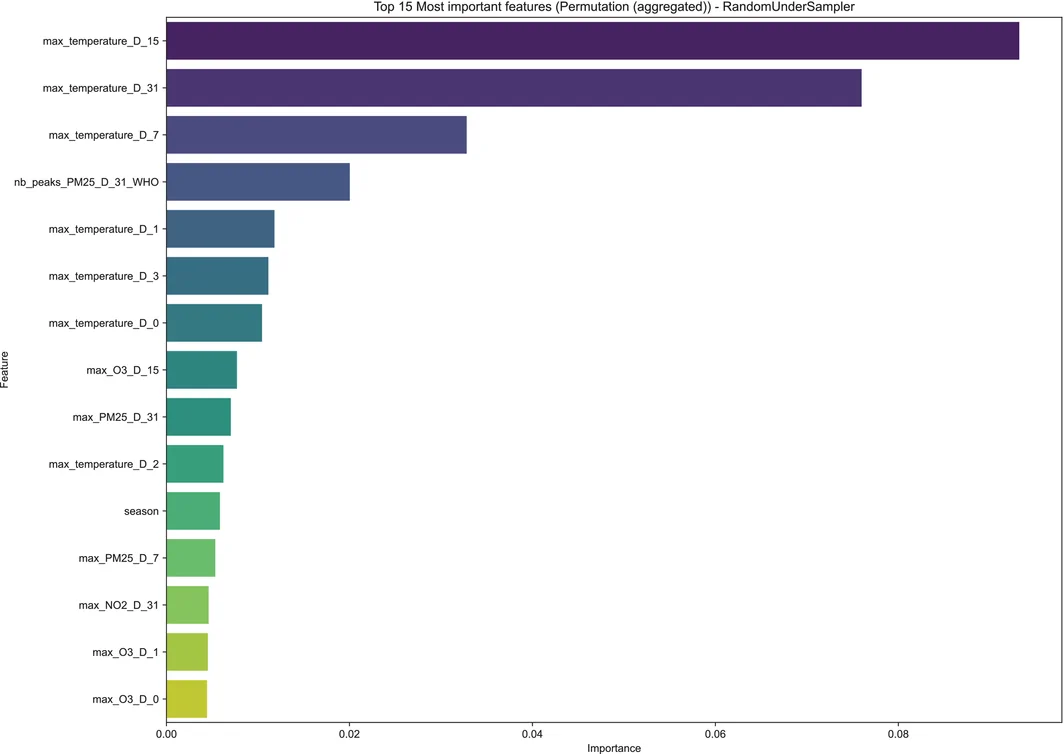
The top 15 main features in the death predictive model.

The 15 most important features for death prediction in the model were high temperatures (maximum temperature in the 15‐day window before death: D15, as well as D31, D7, D3, D0, D1, and D2); PM_2.5_ (the number of peaks above the WHO threshold in the 31‐day window before death, as well as the maximum value of PM_2.5_ reached in the 7‐ and 31‐day windows before death: D7 and D31), PM_10_ (maximum value of PM_10_ in the 7‐day window before death: D7), O_3_ (maximum value of O_3_ in the 1‐day, 15‐day, and 0‐day windows before death: D1, D15, and D0) (Figure 4).

## 4 Discussion

This study presents an innovative geomatic approach linking short‐term air quality at an infra‐communal resolution with mortality in a registry‐based cohort of cancer patients over a three‐year period. The main findings are as follows: (1) Identification of exposure clusters: Unsupervised analysis revealed distinct patient clusters based on air quality exposure; (2) Geographic patterning: Cluster assignment was non‐random and reflected geographic variation in pollution, highlighting urban hotspots; (3) Differential mortality: The four clusters exhibited significantly different mortality rates. Clusters 1 and 4, characterized by the highest levels of particulate matter, showed elevated mortality (12% and 13%, respectively) compared with 9% in the overall cohort, whereas Cluster 3, the least polluted, showed reduced mortality (8%) (chi‐square test, *p* < 0.001); (4) Predictive algorithm: Machine‐learning algorithms enabled the development of a moderately efficient mortality prediction model, achieving a recall of 80%; (5) Key predictors: The most influential air quality variables for predicting death were higher temperature and PM_2.5_ levels over the month preceding death (up to D‐31), suggesting potential windows for preventive interventions.

A key strength of our geomatic approach is the high spatial resolution of particulate matter exposure assigned to each patient. Using our geocoding tool, pollutant data were mapped at a 50 × 50 m granularity, considerably finer than the commonly used communal‐ or city‐level resolution [6, 15, 30]. Recent studies have shown that using finer spatial granularity improves the accuracy of exposure assessment, particularly for PM_2.5_ and its association with mortality. For instance, recent investigations in Russia [31] and Ireland [32] employed a 1 × 1 km resolution, demonstrating the value of high‐resolution data for epidemiological analyses. The achievable spatial resolution depends on the density of ground‐based monitoring stations [32], but can be further enhanced by incorporating satellite‐derived data, as demonstrated in previous studies [33]. The Moran's *I* test applied to the Paris area was statistically significant, indicating that certain geographic locations are more likely to experience specific levels of air pollution. This finding supports the hypothesis that air quality can vary over short distances, underscoring the relevance of our high‐resolution (small granularity) approach [31, 33].

We observed that Clusters 1 and 4, characterized by the highest levels of particulate matter (PM_2.5_, PM_10_, and NO_2_), were associated with increased mortality rates (12% and 13%, respectively) compared with 9% in the overall cohort. In contrast, Cluster 3, with the lowest pollutant levels, was associated with reduced mortality, suggesting a correlation between air pollution exposure within clusters and patient outcomes. Exposure to PM_2.5_ has been consistently associated with increased mortality [13]. Experimental studies, both in vitro and in vivo, have confirmed that air pollutants originating from residual oil combustion and traffic‐related sources are particularly linked to short‐term adverse health effects [13]. Other studies linked air pollution to death in cancer patients [8, 13]. A recent nationwide Brazilian work reported that for every 10 μg/m^3^ increase in 3‐days average PM_2.5_ exposure (granularity of 28 km × 28 km, at the city level), the odds ratio for all‐cancer mortality was 1.04 (95% CI 1.03–1.04) [15]. In our PCA, PM_2.5_, PM_10_ and NO_2_ were associated along the PC1 axis. Correlations between NO_2_ and PM_2.5_ have been already reported in several studies, notably from the ESCAPE study (European Study of Cohorts for Air Pollution Effects) that investigated relationships between outdoor air pollution and health using cohort studies across Europe [34]; and correlations between PM_2.5_ and PM_10_ are also reported [35]. PM_2.5_ and PM_10_ come from traffic, wood heating, industrial plants, agriculture, wildfire smoke and dust, and differ from their size; NO_2_ is mostly an urban air pollutant that comes mainly from traffic. We also found in our PCA that O_3_ and temperature were associated along the PC2 axis. The formation of O_3_ involves a complex interplay between chemical reactions of O_3_ precursors. It has been widely observed that a high temperature is often linked to O_3_ pollution [34, 36, 37]. Some studies report synergistic effects of O_3_ and extreme temperature on mortality (e.g., heat and ozone exposure increased respiratory mortality risk in London, reported in a 2024 study on the 2010–2018 period [38]). A recent analysis has shown that populations worldwide are more exposed to excessive ozone (O_3_) levels when chronic exposure—typically defined as exposure exceeding 90 days—is considered. Short‐term ozone exposure less frequently exceeds high threshold levels [39]. This may explain why O_3_ did not appear among the top five predictors of mortality in our analysis, as the mortality risk linked to long‐term exposure is significantly greater than that associated with short‐term exposure [39]. Regarding long‐term temperature rise and global warming [40], which was not the focus of our study, it is essential for researchers across all disciplines to collaborate in developing practical solutions. Only through interdisciplinary efforts can we tackle the complex challenges posed by global warming and implement effective strategies to mitigate its impact on health, ecosystems, and societies.

Our work reports that confounding factors associated with death were an older age, to be male, and to be affected by thoracic cancer. These findings have already been described in previous studies. 70% of cancer deaths occur in the population of patients > 70 years old [40]. The cancer mortality rate is known to be higher among men than women (171.5 per 100,000 men and 126.3 per 100,000 women) according to the latest 2025 American NIH (National Institutes of Health) statistics [41]. The mortality rate in lung cancer patients is reported to be the highest among cancer patients worldwide by the WHO. Additionally, PM_2.5_ exposure has been reported to be significantly associated with lung cancer mortalities in different works.

The 31‐day lag window in our work was guided by literature which reports a delayed impact of short‐term air quality exposure on deaths [20, 21, 22]. The main air quality features allowing us to predict death in our model were higher temperature and PM_2.5_ level within the whole previous month (until D‐31) before death. A recent Bavarian German work reported as well a strong linkage between high‐mortality events in cancer patients and significantly above‐average levels of particulate matter (PM_2.5_). The authors used the ZIP code to join the patients' data and air quality data, and the air quality data were in the 31 day window before death [42].

Our results suggest that short‐term exposure within the 31 days lag time before death can predict the mortality of cancer patients. This delay gives space for prevention measures and public health policies especially in fragile cancer patients, even though more robust data in the field is needed to complete our work. WHO guidelines are stricter than EEA or French guidelines in terms of air quality thresholds. In our predictive model, air quality data above the WHO thresholds were among the top 15 most important air quality features. This encourages the use of the WHO guidelines to define a better air environment. Current thresholds are used for alerting global population. But for fragile populations such as cancer patients, lower thresholds should be defined to be correlated with the potential medical impact, as it is the case in asthma population [43].

Our study is based on a retrospective study which is one limitation in the interpretation of the results. However, the study was also meant to develop a geomatic approach with statistical tools to correlate air quality data with a specific outcome: death. The main objective has been reached in that sense. Another limitation is the monocentric study and the patients' geographic origin, mainly from Paris itself and the west suburb of Paris. A broader study with more diverse environmental backgrounds, with additional information about professional exposure, would help characterize air quality exposures and its impact on health for other scientific and medical questions.

One important factor to consider in our cohort is the overrepresentation of breast cancer patients, who make up more than 60% of the cohort, as Institut Curie is widely recognized for its management of breast cancer. This selection bias may lead to an underestimation of the overall mortality rate in other cancer localizations, as the cohort predominantly includes breast cancer patients. To mitigate this bias, future analyses should account for this disproportionate representation and its potential impact on the findings. Our study population is predominantly composed of wealthier patients from the western Parisian departments, which could serve as a potential confounder in our analysis. Additionally, the lack of socioeconomic data represents a limitation of the study.

To analyze the impact of air quality on medical outcomes is a new exciting field of research where pollution is probably underestimated. However, to generalize about our methodological approach, first a wide range of non‐health data is now available at fine spatial scales. This study demonstrates how such data can be leveraged, through geomatics, to enhance our understanding of environmental risk factors. Second, in a particularly insightful way, the objective was not to identify the risk factors of cancer themselves, but rather to assess the impact of a degraded living environment—characterized here by temperature and air pollution—on the outcomes of patients already weakened by cancer. Our work represents a truly interesting and unconventional research strategy, offering a novel way to frame the scientific question. The originality of our approach lies in assessing the impact of air quality on the prognosis of cancer patients, in conjunction with the emergence of large‐scale, high‐resolution datasets.

In our study, we focused on the global characteristics of the cohort, considering the overall mortality and air quality exposure patterns across cancer patients without delving into stage‐specific or pathology‐specific differences. This approach allowed us to identify general trends in air quality exposure and its impact on mortality within a broad patient population. However, future studies could benefit from a more detailed analysis, examining how specific stages of cancer or tumor types influence mortality outcomes in relation to air pollution. Such an approach would provide a more granular understanding of the intersection between cancer progression and environmental factors, offering valuable insights for tailored public health strategies.

Future directions of our project would be the integration of air quality data with patients' biological data to understand biological mechanisms in clinical as well as in translational research. Some works already study cancer patients' biology with environmental exposures. For example in the ongoing SPECTA clinical trial (Screening Cancer Patients for Efficient Clinical Trial Access, NCT02834884), sponsored by the EORTC (European Organisation for Research and Treatment of Cancer), a sub‐part of the trial, named RP‐1920 BioRadon, is focusing on the correlation between the molecular profiles of lung cancer patients and indoor radon concentration. Radon is a colorless, odorless radioactive gas formed by the radioactive decay of the small amounts of uranium that occur naturally in all rocks and soils and can be found in materials for house construction at small levels. Similar works with air quality data could be developed. The specific mechanisms by which short‐term PM_2.5_ exposure affects site‐specific cancer death are not yet clear, but biomarkers associated with oxidative stress, inflammatory response, endothelial dysfunction, thrombosis and coagulation, and epigenetic alterations have been reported to be associated with PM exposures [44, 45, 46, 47]. Outdoor air pollution has also been linked to several epigenetic modifications [48], including changes to posttranslational modifications of histones, 5‐hydroxymethylation, and most notably DNA methylation [49]. Our work shows the large possibilities of research in the field of environment and health and the feasibility of it. To develop and structure these geomatic approaches and statistical tools in anti‐cancer centers in France and abroad is a priority.

## Author Contributions

**Loice Pokam:** investigation, methodology, visualization, formal analysis, data curation, writing – review and editing, validation, software. **Joséphine Bocquet:** investigation, data curation, formal analysis, writing – review and editing. **Nadia Hégarat:** conceptualization, writing – review and editing, supervision. **Julie Perlbarg‐Samson:** writing – review and editing, data curation. **Thomas Balezeau:** data curation, writing – review and editing. **Maud Milder:** data curation, writing – review and editing, validation. **Bastien Rance:** writing – review and editing, data curation. **Hector Countouris:** writing – review and editing, data curation. **Julien Guerin:** software, project administration, resources, writing – review and editing. **Flavien Gilles:** writing – review and editing, software, project administration, resources. **Jean Gaudart:** conceptualization, validation, methodology, supervision, writing – review and editing. **Nicolas Girard:** conceptualization, writing – review and editing, writing – original draft, supervision. **Clémence Basse:** conceptualization, supervision, project administration, writing – original draft, writing – review and editing, methodology.

## Conflicts of Interest

The authors declare no conflicts of interest.

## Supporting information


**Table S1:** Variance explained by the first four principal components (PCA).
**Figure S1:** Flow chart.
**Figure S2:** Geographic origin of patients.
**Figure S3:** Features plotted on PC1 and PC2 (PCA). C1 groups all variables related to PM2.5, PM10, and NO2, while PC2 relates to O3 and temperature.
**Figure S4:** Confusion matrix.

## Data Availability

All data used in this project are the property of Institut Curie and can be accessed upon reasonable request addressed to the corresponding author. Further information is available from the corresponding author upon request.
