# Supplementary material for: High‐Throughput Geocoding to Assess Short‐Term Air Pollution in Correlation With Mortality in a French Cancer Patient Cohort
Source: Int J Cancer. 2026 Jun 7;159(8):2054–65. doi: 10.1002/ijc.70589 (PMC13495927; doi:10.1002/ijc.70589)

High-throughput geocoding to assess short-term air pollution in correlation with mortality in a French cancer patient cohort

Loice Pokam, Joséphine Bocquet, Nadia Hégarat, Julie Perlbarg-Samson,
Thomas Balezeau, Maud Milder, Bastien Rance, Hector Countouris, Julien Guerin, Flavien Gilles, Jean Gaudart, Nicolas Girard, Clémence Basse

Table of Contents

Sup Table 1: Variance explained by the first four principal components (PCA) p3

Supplemental Figure 1: Flow Chart p4

Supplemental Figure 2: Geographic origin of patients p5

Supplemental Figure 3: Features plotted on PC1 and PC2 (PCA). C1 groups all variables related to $PM_{2.5}$, PM_{10}, and NO_2, while PC2 relates to O_3 and temperature. p6

Supplemental Figure 4: Confusion matrix p7

Supplemental Table 1. Proportion of variance explained by the first four principal components in the PCA

Principal Component	Percentage of explained variance (%)	Cumulated variance (%)
PC1	26.33	26.33
PC2	17.54	43.87
PC3	9.84	53.71
PC4	8.17	61.17

Supplemental Figure 1: Flow Chart

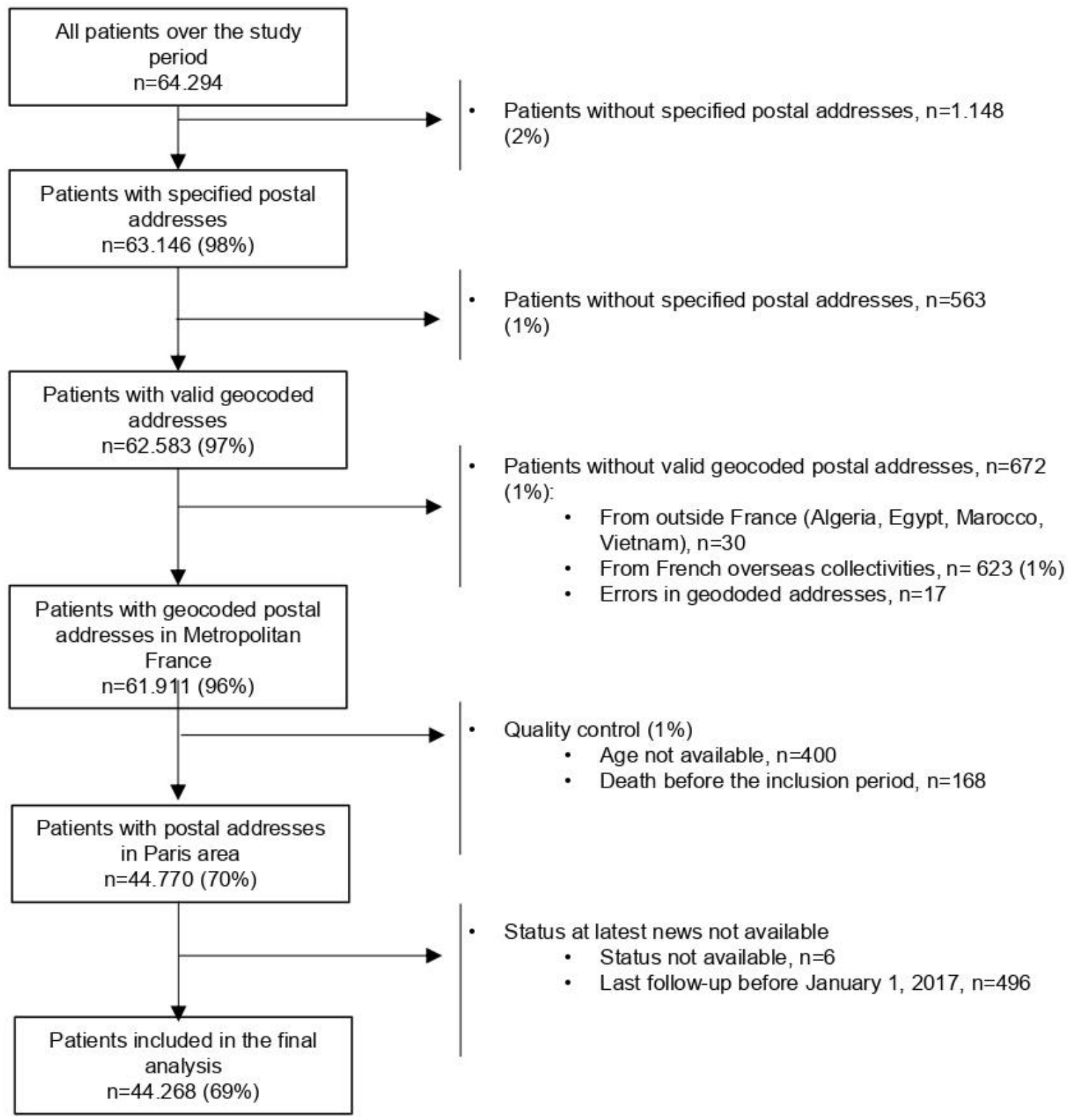

Supplemental Figure 2: Geographic origin of patients

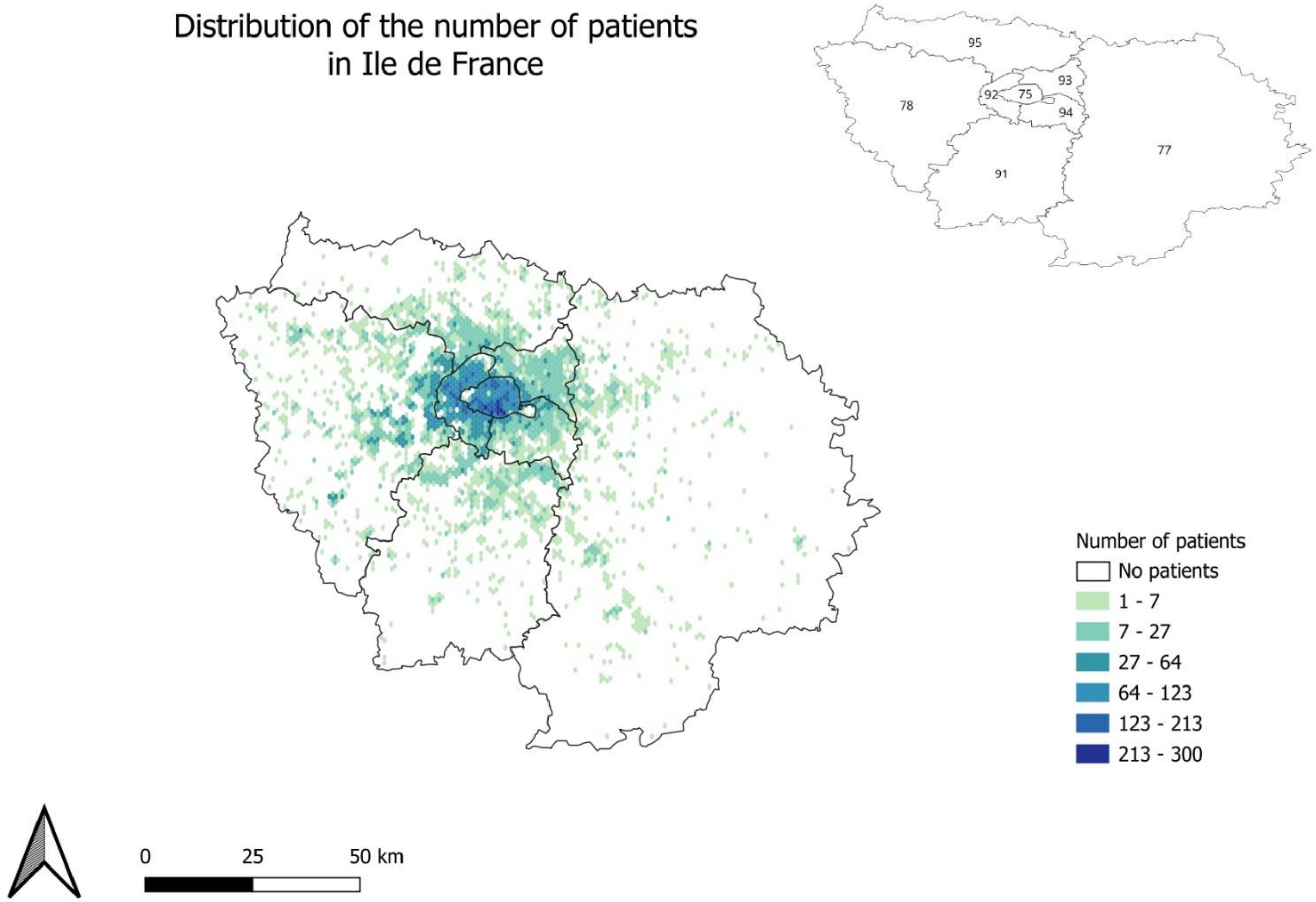

Supplemental Figure 3: Features plotted on PC1 and PC2 (PCA). C1 groups all variables related to $PM_{2.5}$, PM_{10}, and NO_2, while PC2 relates to O_3 and temperature.

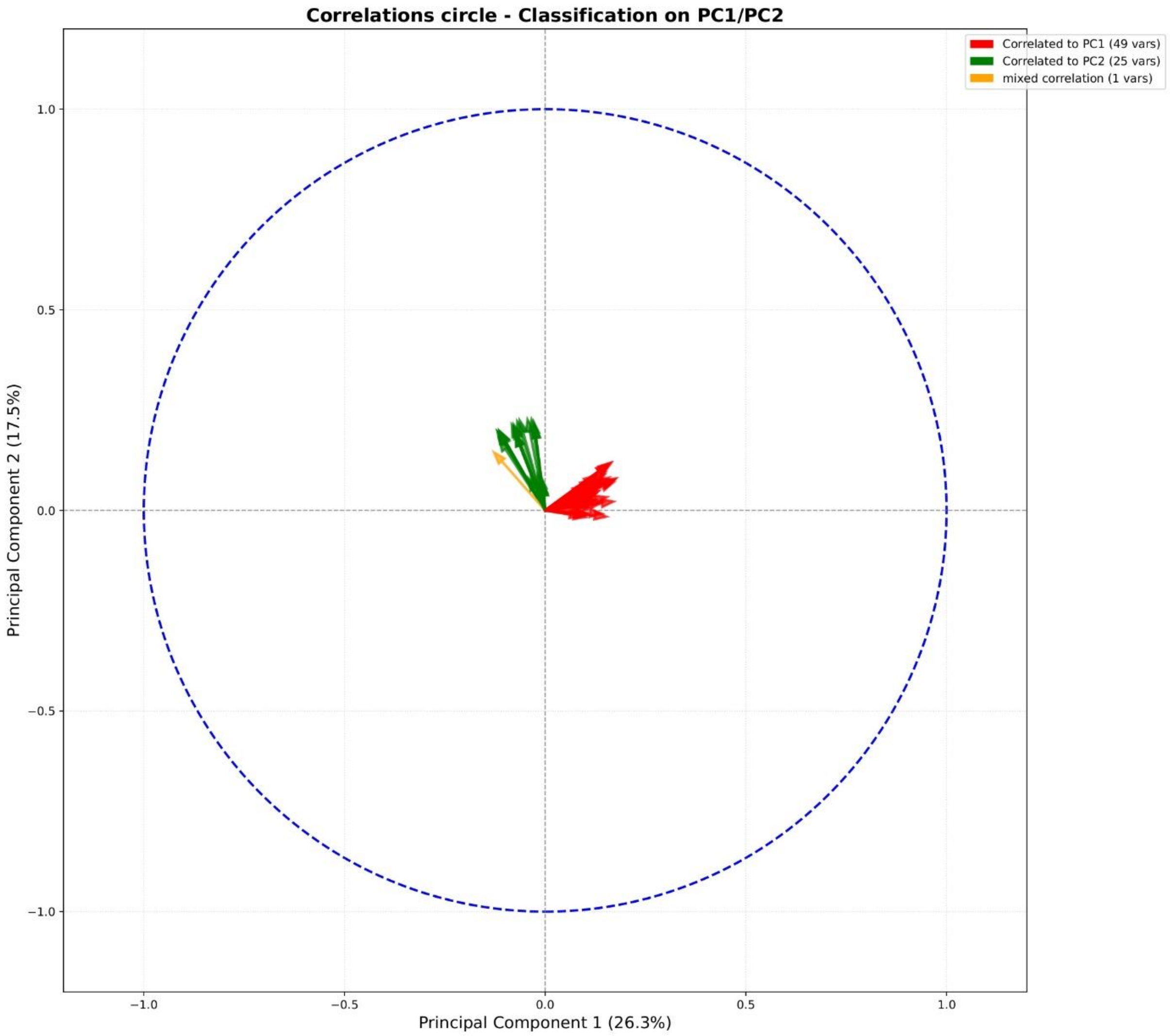

Supplemental Figure 4: Confusion matrix

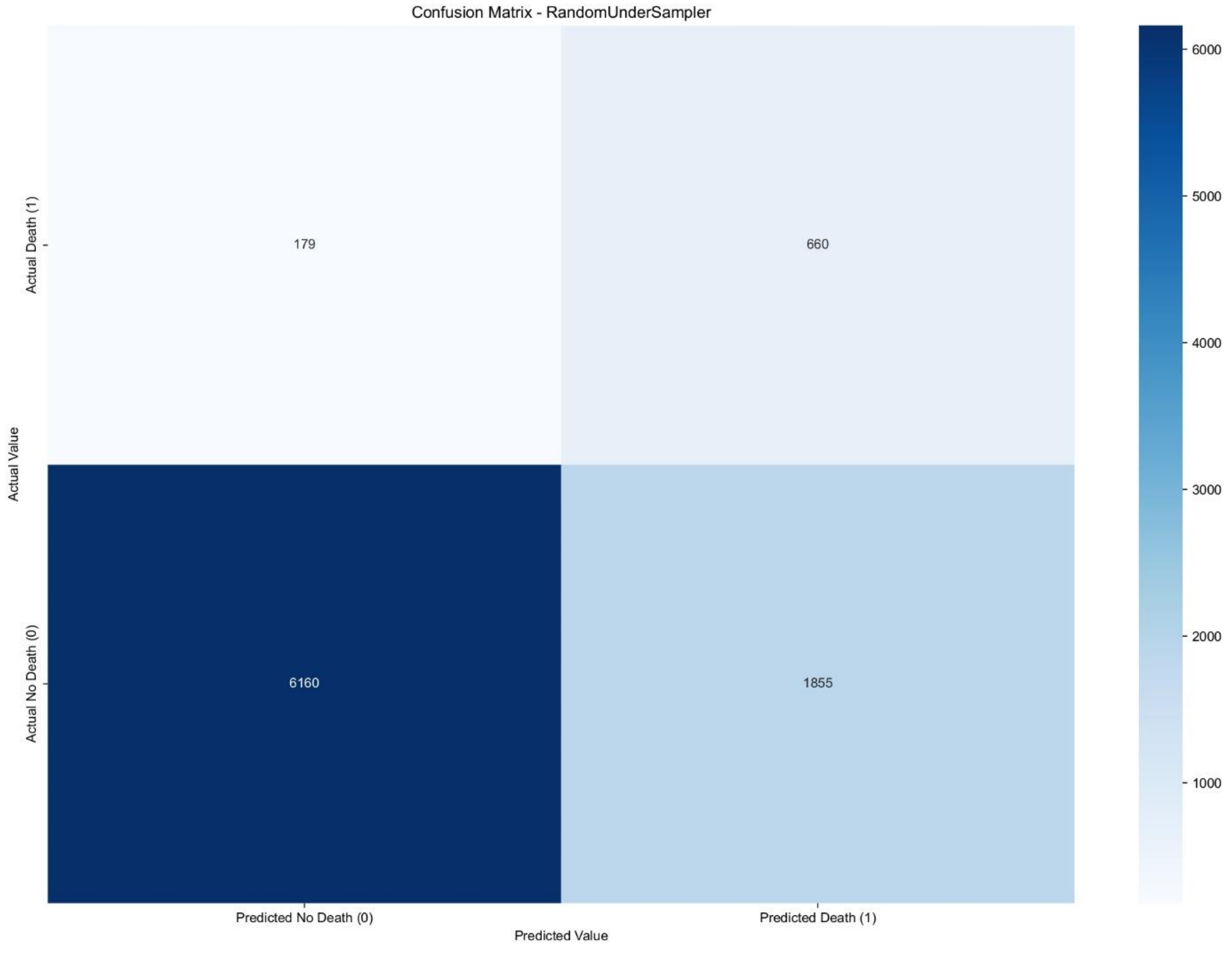

Supplement: Supplementary file 1 [file IJC-159-2054-s001.pdf]
